# Complete Annotated Genome Sequence of the Salmonella enterica Serovar Typhimurium LT7 Strain STK003, Historically Used in Gene Transfer Studies

**DOI:** 10.1128/MRA.01217-20

**Published:** 2021-03-11

**Authors:** Julie Zaworski, Anne Guichard, Alexey Fomenkov, Richard D. Morgan, Elisabeth A. Raleigh

**Affiliations:** a Research Department, New England Biolabs, Ipswich, Massachusetts, USA; University of Maryland School of Medicine

## Abstract

The genome of Salmonella enterica serovar Typhimurium LT7 comprises a chromosome and two plasmids. One plasmid is very close to pSLT of *Salmonella* Typhimurium LT2; the second harbors a shufflon region. Prophage content is distinct: LT7 lacks Fels-1, while Gifsy-1 and Fels-2 show island-like divergence and likely programmed inversion, respectively.

## ANNOUNCEMENT

The isolates Salmonella enterica subsp. *enterica* serovar Typhimurium LT7 and LT2 were early model organisms for the study of gene transfer (1–3). *Salmonella* taxonomy has varied (4); these two lysotypes differ in prophage content (5). Isolates of LT2 were found to be more genetically stable than those of LT7 (6), which remains a model for its mutator properties (6, 7). Our LT7 isolate, STK003, was MST1656 from Stanley Maloy.

A Pacific Biosciences RS II instrument and the SMRT Analysis pipeline were used for sequencing, contig assembly, and modification analysis as described in reference 8. Briefly, cells were grown in LB and harvested, and DNA was then isolated with phenol-chloroform (9). A SMRTbell library was constructed from 5 µg sheared (Covaris g-TUBE, 5 to 10 kb) DNA; sequencing used polymerase 6, chemistry 4. A total of 1,964 Mb of sequence in 131,952 polymerase reads with mean subread lengths of 3,046 bp and an *N*_50_ subread length of 4,016 bp was obtained (∼329× coverage). Assembly (RS_HGAP_Assembly.3; smrtanalysis_2.3.0.140936.p5.167094) specified a 5-Mb expected genome size, minimum subread length (MSL) of 1,000 bp, minimum read accuracy of 0.80, and minimum polymerase read length (MPRL) of 1,000 bp (other parameters were at default settings). Three contigs resulted with sizes of 4,835,584 bp, 115,234 bp, and 109,793 bp. The overall structure was rechecked with RS_HGAP3, resulting in an MSL of 3,000 bp and an MPRL of 9,000 bp.

Annotation, manipulation, and analysis employed Geneious_9.1.8 (Biomatters) with the options “Annotate_from_Database” (resulting in a 100% DNA match to the genome sequences found under the GenBank accession numbers AE006468, AE006471, and NZ_CP012929), “Sequence,” and “change residue numbering.” Circularization employed reference 10 or the procedure described in reference 11, File S1. Coordinates were set to agree with the reference origins. The final circular polished (Quiver) assemblies were a chromosome (4,817,454 bp) and two plasmids (93,946 bp and 88,171 bp). The GC contents are, respectively, 52.2%, 53.1%, and 50%.

**FIG 1:**
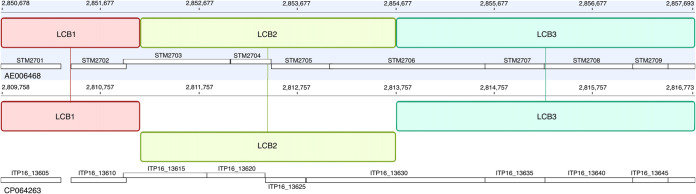
Inversion of candidate phage tail fiber coding sequence of Fels-2. Colored blocks represent segments of matching sequence (LCB), with genome coordinates above the blocks. Blocks above the horizontal lines have identity on the same strand; for the one below it, the bottom strand is identical to the top strand of its homolog. Accession numbers are below the horizontal lines; below that are open boxes labeled with locus tags of predicted coding DNA sequences (CDS). CDS STM2702 of LT2 (GenBank accession number AE006468) and its identical homolog, ITP16_13610 (*fin*) of LT7 (CP064263), adjoin the inversion. Alternative C-terminal segments are fused to identical N termini for STM2703 and ITP16_13615; similarly, identical N termini of STM2706 and ITP16_13630 are fused to alternative C termini. (We observe that notes in the LT2 sequence misleadingly describe STM2703 as similar to *pin* of e14; a more extensive description of STM2702 accurately notes similarity to site-specific recombinases and invertases.)

The 4.8-Mb chromosome of STK003 shows 98.8% nucleotide identity to LT2 (MAFFT [12]). One plasmid is a close relative of pSLT of LT2; the second most closely resembles p12-4374_96 from Salmonella enterica subsp. *enterica* serovar Heidelberg (GenBank accession number NZ_CP012929). LT7 lacks prophage Fels-1, which is expected from the lysotype (13, 14). Prophages Fels-2 and Gifsy-1 of the two strains differ in important respects. Gifsy-1 carries a substitution of ∼7.5 kb between LT2 loci STM2620 and STM2630, likely from a different prophage (e.g., serovar Newport [GenBank accession number CP025230]). In contrast, Gifsy-2 is almost identical to the LT2 copy. Inversion of a Fels-2 tail fiber gene (Fig. 1; from the Geneious_Prime_2020.2.4 implementation of Mauve [15]) likely was mediated by *pin* homolog ITP16_13610 (LT2 locus tag STM2702) (16). Alternative host ranges are conferred by similar inversions mediated by *cin* of phage P1 and *gin* of phage Mu (17) and possibly *pin* of the e14 prophage-like element. The second plasmid harbors an ∼2-kb region related to shufflons of IncI conjugal plasmids, which employ site-specific recombination to generate variation in pilus protein adhesion specificity (18, 19).

The methylation phenotype (from RS_Modification_and_Motif_Analysis) and genotype agree with activities expected for the serovar (Table 1), confirming functionality of methyltransferase (MTase) open reading frames (ORFs). No major single nucleotide polymorphisms (SNPs) were found in the restriction-modification (R-M) systems. Partial activity of M.SenLT2IV orthologs was previously reported for LT2 and other Salmonella enterica serovars (20).

**TABLE 1:** Methylation specificity of DNA MTases in Salmonella enterica subsp. *enterica* serovar Typhimurium LT7^*a*^

MTase^*b*^	Motif^*c*^	Type of modification	Sites with IPD > 2 (%)^*d*^
M.SenLT7I (StyLT)	5′ CAG*AG 3′	m6A	99.2
M.SenLT7III (StySA)	5′ GATC*AG 3′	m6A	99.3
M.SenLT7II (StySB)	5′ G*AGNNNNNNRTAYG 3′ 3′ CTCNNNNNNY*ATRC 5′	m6A	98.8 100
M.Dam	5′ G*ATC 3′	m6A	99.7
M.Dcm	5′ C*CWGG 3′	(m5C)	0.3
M.SenLT7IV	5′ ATGC*AT 3′	m6A	54.9

a The PacBio technology does not detect m5C accurately.

b For MTases associated with restriction activities (R-M), synonyms found in the restriction literature are in parentheses.

c Modified sequence motifs (* precedes the methylated base).

d The percentage of recognition sites that were methylated. IPD, interpulse duration (23).

### Data availability.

The genome sequences described here have been deposited under GenBank accession numbers CP064263 to CP064265 (assembly number ASM1547561v1) (PGAP annotated [21, 22]) and SRA numbers SRR12788408 and SRR12788409.
